# Comparative Clinical Outcomes in the Treatment of Anterior Crossbite Using Three Different Appliances: A Case Series

**DOI:** 10.7759/cureus.68703

**Published:** 2024-09-05

**Authors:** Guru Vishnu, Ramesh R

**Affiliations:** 1 Pediatric and Preventive Dentistry, Saveetha Dental College and Hospital, Saveetha Institute of Medical and Technical Sciences, Saveetha University, Chennai, IND

**Keywords:** anterior crossbite, children, interceptive orthodontics, malocclusion, mixed dentition

## Abstract

Anterior crossbite, a misalignment of the upper front teeth in which the bite is behind the lower front teeth, is a common dental condition in mixed dentition. This case series examines the clinical results of three orthodontic appliances, namely Catlan’s appliance, the removable finger-spring appliance, and the 2x4 appliance, in the management of anterior crossbite. The first case study involves a seven-year-old patient with a single-tooth crossbite. The patient was treated using a 2x4 appliance, which resulted in a satisfactory correction of the problem within a reasonable treatment duration. The therapy was comfortable for the patient and resulted in high patient satisfaction levels. The second case involves a nine-year-old patient with a crossbite on both of their permanent central incisors. The patient was treated using a Catlan’s appliance, which had the shortest treatment duration but resulted in the highest level of discomfort. The third case study focuses on a nine-year-old patient with a single-tooth crossbite. The condition can arise from dental or skeletal disparities and, if not managed, can cause periodontal damage, aberrant enamel erosion, and temporomandibular joint disorders. Prompt intervention using various appliances is essential for rectifying the crossbite and averting potential consequences. This study uniquely compares Catlan’s appliance, the removable finger-spring appliance, and the 2x4 appliance, each distinct in design and application, and evaluates a range of outcome measures, including treatment duration, occlusal improvement, patient comfort, and adverse effects. All three appliances, such as Catlan’s appliance, the removable finger-spring appliance, and the 2x4 appliance, are effective in correcting anterior crossbite, demonstrating that multiple treatment options can achieve successful outcomes.

## Introduction

Anterior crossbite is a dental condition in which one or more of the upper front teeth bite behind the lower front teeth [1]. Crossbite involving a single tooth is the most common issue during the early stages of mixed dentition. Once the crossbite is diagnosed, the treatment plan should be straightforward and noninvasive, requiring little chairside time, minimally involving the patient, and quickly fixing the crossbite without damaging the surrounding structures. Several authors have proposed a variety of treatment options, including fixed and removable appliances, Hawley retainer with double cantilever springs, labial and lingual archwires, Catlan's appliance (lower inclined bite plane), and crowns made of composite material or stainless steel.

Early intervention is crucial for correcting anterior crossbite to prevent the development of more severe orthodontic and skeletal problems later in life. Various treatment protocols exist for addressing this condition, each with its advantages and limitations. In young children, patient compliance with a removable item can vary. Anterior crossbite can be classified into skeletal and dental categories. In skeletal anterior crossbite, the issue stems from the jawbone, while dental anterior crossbite is caused by the improper positioning of the teeth [2,3].

The prevalence of anterior crossbite in mixed dentition, which includes both primary and permanent teeth, is a common clinical observation. With a documented prevalence of 4%-5%, it typically manifests in the early stages of mixed dentition [4]. Since the tooth is in its active stage of eruption and the root is still forming, the best age range for correction is immediately as soon as it is diagnosed. If left untreated, anterior crossbite can lead to a range of complications, including abnormal wear of the teeth, periodontal issues, temporomandibular joint (TMJ) disorders, and aesthetic concerns [5,6].

This case series is unique because it offers a direct comparison of factors such as the type of appliance (fixed or removable), components, treatment duration, activation, gingival health, patient compliance, discomfort, and cost of three different orthodontic appliances used to treat anterior crossbite: Catlan's appliance, the removable finger-spring device, and the 2x4 appliance. In contrast to several studies that focus on individual appliances or more general comparisons, this case series presents a thorough examination of these three techniques, offering insightful information about their relative efficacy. Through the assessment of treatment outcomes in various aspects, including treatment time, occlusal improvement, patient comfort, and adverse effects, this research provides a thorough understanding of the clinical acceptance and performance of each appliance.

The correction of anterior crossbite in mixed dentition is essential to prevent developing dental and skeletal complications. Various appliances, including the 2x4 appliance, Catlan's appliance, and the posterior bite plane with Z-spring, offer different mechanisms and benefits for achieving proper occlusal alignment. This case series presents the efficacy and clinical factors to consider when using these treatment methods, with support from contemporary scientific research. This case series was written following the CARE guidelines (2013) for reporting case reports.

## Case presentation

Case 1

A nine-year-old patient presented to the Department of Preventive and Pediatric Dentistry with a chief complaint of backwardly placed upper front teeth. Extraoral examination revealed a symmetrical bilateral appearance and a convex profile. Intraoral examination identified a crossbite involving the maxillary right central permanent incisor. The patient did not have a history of trauma or a family history of class III malocclusion. However, a retained deciduous tooth, which was extracted two years ago, potentially led to the development of the crossbite.

Diagnosis

Angle’s Class I malocclusion with anterior crossbite in relation to 11 (right permanent central incisors). 

Treatment plan

**Figure 1:**
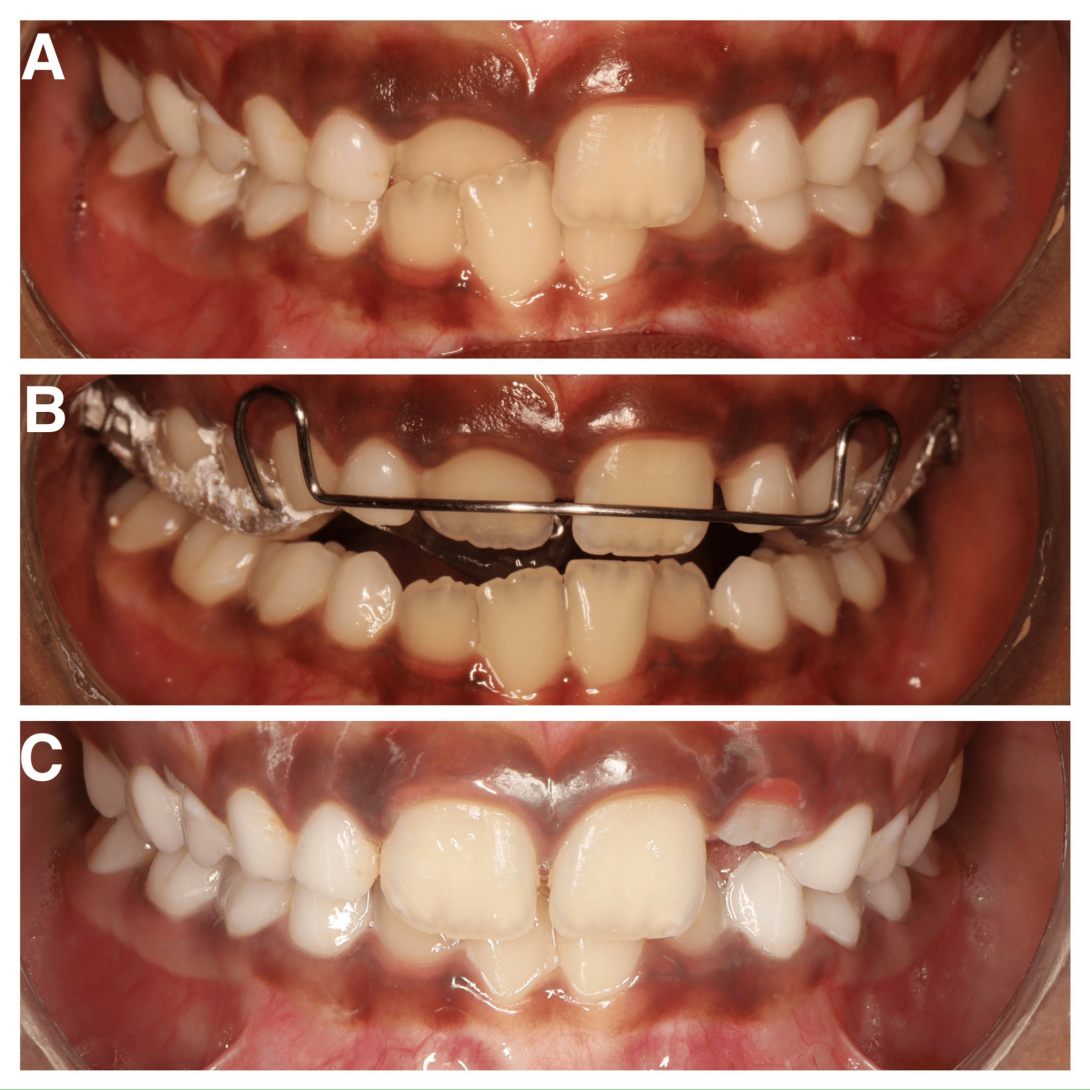
(A) Pre-treatment photo; (B) insertion of removable Z-spring with posterior bite plane; (C) post-treatment photo.

An impression was taken using alginate, resulting in the creation of two casts: one for practical use (working model) and another for examination and analysis (study model). Moyer's mixed dentition analysis revealed that 0.3 mm of space would be available in the maxillary arch for the alignment of the permanent incisor. This ensures that there will be enough area for excellent occlusion and alignment. The lateral cephalometric scan did not indicate any skeletal anomalies in the maxillary or mandibular arches. A dental appliance called the Hawley appliance, which includes a Z-spring and a posterior bite plate, was created. The retentive components of Adam's appliance were the clasp and labial bow, both made from 21-gauge circular stainless-steel wire. The Z-spring was the active component of the appliance, responsible for exerting force on the central incisor to move it within the arch. The stabilization of these was achieved by using a self-cure acrylic resin (Figure 1). A posterior bite plane was added to prevent contact between the mandibular incisors and the anterior teeth, thus correcting the anterior crossbite. The appliance was activated by gradually activating the active components in a labial direction. Subsequently, the Z-spring arms were placed in the mouth, ensuring they were in contact with and parallel to the palatal surfaces of the central incisor affected by the crossbite. This arrangement helped in moving the affected teeth labially. The patient then received instruction on how to properly insert and remove the appliance, as well as instructions to wear it consistently, including while sleeping, until they achieved the desired correction. It was instructed to the patient to remove the appliance before meals. For eight weeks, the Z-springs were triggered weekly. The achievement of a complete alignment of the upper and lower incisors, resulting in a class I molar relationship, demonstrates the successful repair of the crossbite in an additional two weeks.

Case 2

A nine-year-old patient presented to the Department of Preventive and Pediatric Dentistry with a primary concern of protruding lower anterior teeth. The study of the external features of the face revealed a balanced and symmetrical appearance on both sides with a convex profile. On intraoral examination, it was found that the mandibular incisors (31, 32, and 41) were placed forward and the maxillary central incisors (11 and 21) were in crossbite. The patient had no history of physical injury, and there was no familial occurrence of class III malocclusion.

Diagnosis

Angle’s Class I malocclusion with anterior crossbite in relation to 11 and 21 (right and left permanent central incisors).

Treatment plan

**Figure 2:**
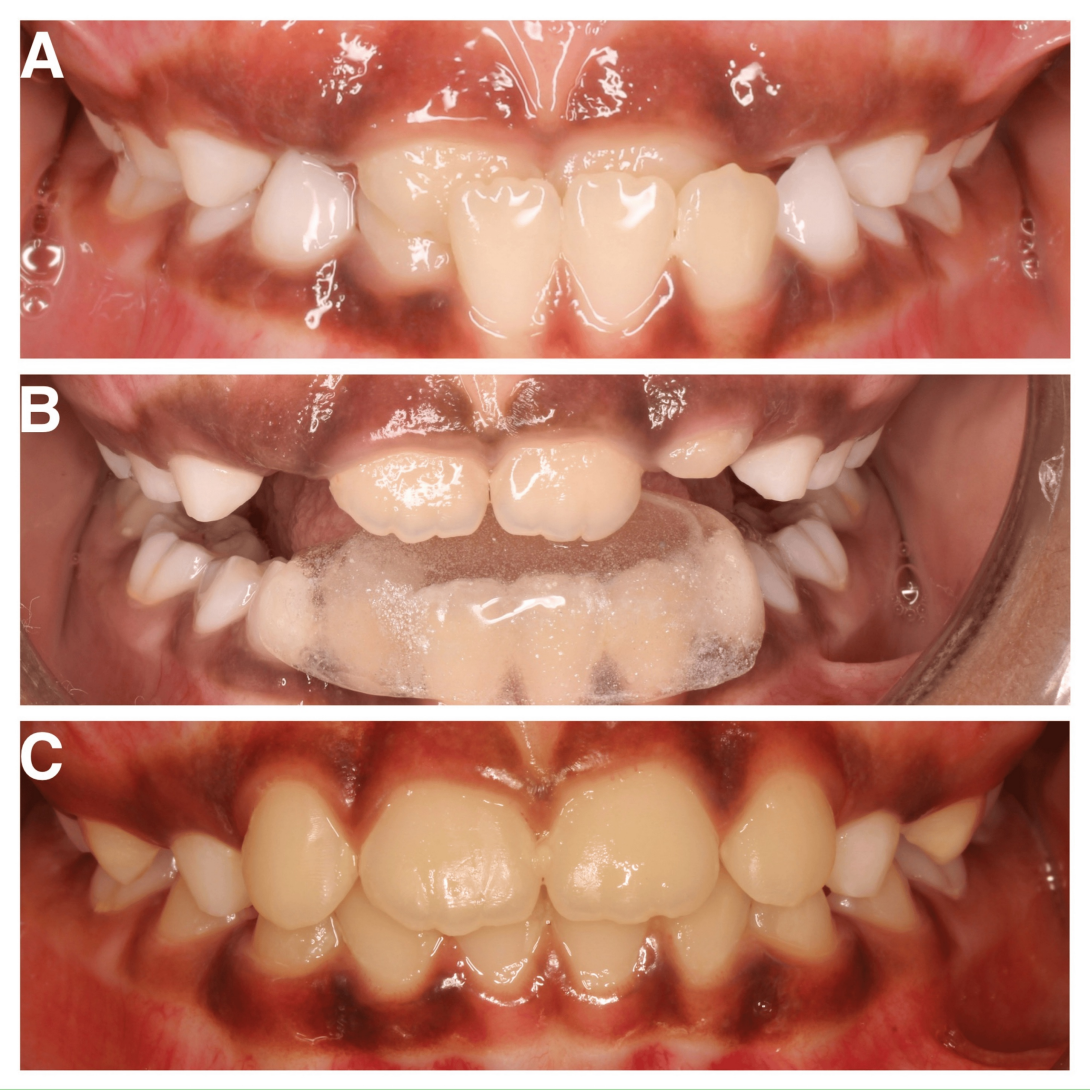
(A) Pre-treatment photo; (B) insertion of Catlan's appliance; (C) post-treatment photo.

Impressions of the arches were made using putty to create study casts. Moyer's mixed dentition analysis determined that there was enough space in the dental arch to allow the teeth to realign. The predicted space availability in the maxillary arch for the alignment of permanent incisors was within the range of 7.5 mm, ensuring adequate room for proper occlusion and alignment. The patient displayed a class I molar relationship, with a 1 mm reversed overjet and a 2 mm overbite. The lateral cephalometric scan revealed no skeletal abnormalities in the maxillary or mandibular arches. The patient was in a growing phase and had mixed dentition with enough space in the maxillary arch to accommodate the malpositioned teeth and the expansion of the arch. After discussion with the parents, a Catlan’s appliance was fabricated at a 45-degree angle to align with the long axis of the upper permanent teeth, and zinc phosphate cement (Pyrex, India) was employed to fix the inclined plane to the mandibular anterior teeth (Figure 2). Following cementation, the incisor area was the only site of contact in an occluded state. The patient was advised to practice proper oral hygiene and returned once a week to assess the treatment's clinical effectiveness. The parents and the patient were notified of the inconvenience that may arise during the operation of the appliance and were encouraged to adapt to a new bite and consume soft meals for the initial few days. The crossbite was successfully adjusted with Catlan's appliance within two weeks. The child will eventually adapt to his or her odd bite, the parents were informed, and during the first few days following cementation, a softer diet was advised. After the crossbite was corrected, the Catlan's appliance was removed. Then, the enamel surface was polished, and topical fluoride (APF gel) was administered. Since the appliance remained in place during the follow-up exams, there was no need for reinforcement. Upon review three months later, no recurrence of the crossbite was detected in the maxillary central incisors.

Case 3

A seven-year-old patient arrived at the Department of Preventive and Pediatric Dentistry with a significant concern about a misaligned front upper tooth. There was no pertinent family or medical history. The assessment of the external features revealed a symmetrical bilateral appearance with a convex profile. The intraoral examination showed a crossbite in the permanent left central incisor. He had a history of trauma two years ago that affected his primary left central incisor. He exhibited a class I molar relation.

Diagnosis

Angle's Class I malocclusion with anterior crossbite in relation to 21 (permanent left central incisor).

Treatment plan

**Figure 3:**
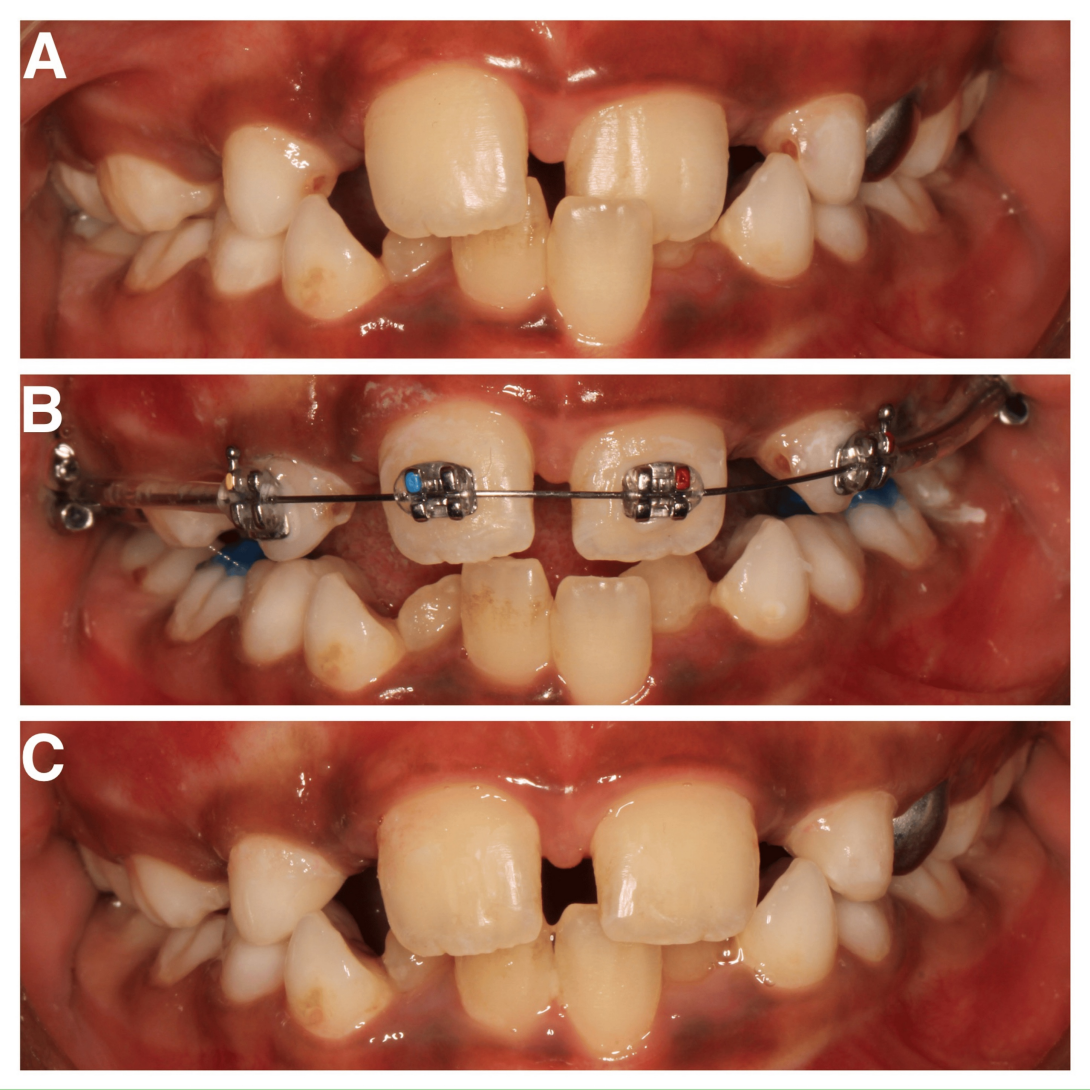
(A) Pre-treatment photo; (B) insertion of 2 by 4 appliance; (C) post-treatment photo.

Impressions of both arches were made using putty to create study casts. Moyer's mixed dentition analysis revealed that the maxillary arch had excess space of 14 mm between the length of the arch and the amount of tooth material present. The analysis revealed sufficient space within the dental arch to facilitate tooth repositioning. The lateral cephalometric scan revealed no skeletal abnormalities in the maxillary or mandibular arches. After consulting with the parents, we decided to use a brief wire-fixed orthodontic treatment that involves employing four preadjusted MBT brackets with a 0.022-inch slot. Since the permanent lateral incisors had not yet erupted, the brackets were placed on the buccal surfaces of both the permanent central incisors and two primary canines. A 0.014-inch round nickel-titanium (Ni-Ti) archwire was cut symmetrically on both sides of the centreline and placed into the slots of the brackets. The wire was ligated using orthodontic modules. A 2-mm thick layer of composite material (Blue Bite-Block, Waldent, India) was applied to the occlusal surface of the patient's permanent first lower molar to elevate the bite. The patient received weekly evaluations, and after three weeks, the incisor teeth exhibited a noticeable forward overjet. The two central permanent incisors were properly positioned. Subsequently, the brackets were detached, and the bite-raising composite was eliminated. Following the removal of the appliance, the enamel was polished, and a layer of topical fluoride was administered. The patient was reviewed for three months (Figure 3).

## Discussion

Interceptive treatment is fundamental in reducing the severity of a developing malocclusion. An old orthodontic saying asserts, "The best time to treat a crossbite is the first time it is seen" [7]. Therefore, when anterior crossbite is accurately detected and corrected, it is a conservative technique for pediatric patients. Anterior crossbite should be intercepted and treated without delay because it is a self-perpetuating condition that, if not treated early, has the potential of growing into skeletal malocclusion and might at a later stage require major orthodontic treatment combined with surgical intervention [8]. Interceptive orthodontic treatment at an early stage can not only boost a young child’s self-esteem but also avoid the need to undergo cumbersome orthodontic treatment in the future [9].

The optimal time to treat an anterior dental crossbite is during the active stage of tooth eruption, which occurs between the ages of 8 and 11. The age of the child is significant, but so is the need for treatment [4,10]. Gender disparities also exist; females are more eager to receive treatment than males. It has been suggested that various criteria, including the child's age, the number of teeth that need to be relocated, the occlusion status, and the motivation of both the child and parents, should be considered when selecting an approach, and doctors occasionally run into difficulties when making this decision [11].

The correction of anterior crossbite in mixed dentition can be effectively managed using various orthodontic appliances, each offering distinct advantages, tailored to patient needs. A dental crossbite does not involve the basal bone; instead, it entails the localized tilting of one or more teeth [12]. Ghodasra and Brizuela state that to treat an anterior crossbite, sufficient space must first be opened, and then the misplaced tooth or teeth must be brought into the proper position across the occlusion [13]. 

An overview of the various factors involved in the treatment provided using the three appliances is mentioned in Table 1.

**Table 1:** Overview of the various factors involved in the treatments provided. Ni-Ti: nickel-titanium.

Feature	2x4 Appliance	Catlan's appliance	Posterior bite plane with Z-spring
Type of appliance	Fixed appliance	Fixed appliance	Removable appliance
Components	Orthodontic brackets, Ni-Ti wire	Acrylic inclined plane at 45 degrees on mandibular anterior	Hawley appliance and posterior bite plane
Treatment duration	Three weeks	Two weeks	Eight weeks
Activation	Activated is not required. There was no need of change of wire	No activation	Z-spring to be activated once every week
Gingival health	Slight inflammation due to plaque development from accumulation of food around brackets	Slight inflammation due to food lodgment in the border of the acrylic base of the appliance	Good gingival health as the appliance is removable and regular oral hygiene can be maintained
Review and recall	One week	One week	One week
Patient compliance	Less compliance required	Less compliance required	High compliance needed for adjustments
Patient comfort	Pricking feeling in the buccal mucosa. Difficulty during brushing	Slight burning sensation after cementation of appliance using zinc oxide eugenol	Uncomfortable due to bulk of the appliance. Interference with speech
Cost	High	Low	Moderate

A prominent method is the 2x4 appliance, which employs brackets and wires on the four upper and lower anterior teeth. A study by Dowsing and Sandler demonstrated the efficacy of this approach, particularly in young patients, by providing precise control over tooth movement and facilitating early intervention during mixed dentition stages [14]. A clinical evaluation by Prakash and Durgesh found significant improvements in patients with mild to moderate anterior crossbites, emphasizing its suitability as a less invasive alternative to fixed orthodontic appliances [9]. Offering the flexibility of adjustment, this appliance can be customized to apply the appropriate force needed for dental correction [15]. Therefore, diastemas, rotations, and improper inclinations of teeth can be treated easily and quickly using this technique [14]. Practitioners must consider what should or should not be treated at the mixed dentition stage. Many malocclusions will self-correct once the transition is complete. The 2x4 appliance, used following trauma, required three weeks of treatment and three follow-ups, causing minimal pain and achieving the highest patient satisfaction, despite higher Gingival and Oral Hygiene Index scores in our case series.

The posterior bite plane with Z-spring is another appliance discussed in the literature for the correction of anterior crossbite. The 2012 study by Bindayel highlighted its effectiveness in disoccluding the anterior teeth, which facilitates their movement into the correct position without interference from the posterior teeth [16]. This appliance is particularly beneficial for minor crossbites and can be easily adjusted to provide the necessary corrective force. The posterior bite plane with Z-spring, designed for retained deciduous teeth, required the longest duration of eight weeks and six follow-up visits, resulting in minimal pain but only average patient satisfaction in our case series.

Catlan’s appliance works on the principle of Newton’s third law of motion; the resin slope functions to tip an anterior tooth labially while the mandibular tooth is tipped slightly in the lingual direction. The early stage of mixed dentition provides an optimal context for reversing the bite with Catlan's appliance. The practitioner must first differentiate between crossbites of dental origin and those of skeletal origin to employ this appliance. The chosen course of action should be comfortable for the child, not harm nearby tissues, correct the crossbite quickly, and not impede the child's ability to grow and develop. Because Catlan's appliance requires little patient involvement and is inexpensive, quick, and simple to build, it was used with the patient in the aforementioned situation. The inability to speak clearly, difficulty eating food, and the possibility of anterior open bite if the appliance is cemented for longer than six weeks are the disadvantages of Catlan's appliance (lower inclined bite plane). As a result, it is crucial to check on the patient every week and make an informed decision to remove the device if treatment takes a long time [17]. Catlan’s appliance, intended for developing crossbite, had the shortest treatment duration of two weeks and only two follow-ups but caused the most dissatisfaction due to pain and appliance dislodgement in our case series.

Removable appliances, such as Hawley retainers and clear aligners, have also been used for crossbite correction. A study by Ulusoy and Bodrumlu indicated that removable appliances are effective for minor to moderate anterior crossbites, offering the advantage of better oral hygiene maintenance and comfort for the patient. However, these appliances require high patient compliance to achieve optimal results [18]. In this case, our therapeutic choice was directed towards a removable finger-spring appliance because there was sufficient space for labialization of the incisors, vertical overbite was less than 1/2 from the length of the crown, and the crossbite was of dental origin. A posterior bite plane was inserted to allow the crossbite correction. This removable appliance is economical and harmless to soft tissues; it maintains proper oral hygiene, reduces chairside time, and is not as bulky as removable appliances with screws. However, the success of therapy depends on patient cooperation. In this case, the removable finger-spring appliance was tolerated. Thus, the bite was reversed in a brief period of four weeks without any damage to the tooth or the periodontium.

## Conclusions

The choice of appliance for anterior crossbite correction should be based on a comprehensive evaluation of the patient's specific dental condition, age, and compliance. The 2x4 appliance, Catlan's appliance, the posterior bite plane with Z-spring, removable appliances, and functional appliances each offer unique benefits that can be leveraged to achieve optimal treatment outcomes. Further comparative studies and long-term follow-up clinical trials are essential to refine these techniques and enhance their efficacy in clinical practice.
